# A scoping review of how behavioural theories, models and frameworks have been applied to the design, conduct, analysis or reporting of clinical trials

**DOI:** 10.1186/s13063-025-08808-8

**Published:** 2025-03-25

**Authors:** Taylor Coffey, Naomi Young, Katie Gillies

**Affiliations:** Aberdeen Centre for Evaluation, University of Aberdeen, Foresterhill, UK

**Keywords:** Behavioural science, Clinical trials, Trials methodology, Scoping review

## Abstract

**Background:**

Clinical trials provide much of the evidence that guides decision making about treatment and care but are often complicated to deliver. Trials can be thought of as complex systems with interacting individuals, as such behavioural science is a useful lens to investigate how trial processes may be improved. To guide selection of an appropriate behavioural theory, model, or framework to further enhance trial processes, we sought to map their past application within the design, conduct, analysis and reporting of clinical trials.

**Methods:**

A scoping review to investigate the breadth of trials methods research that has utilised a behavioural theory, model, or framework was conducted through a database search and citation analysis. Publications investigating any part of the trial lifecycle (from design to reporting) through a behavioural lens were included. Data were extracted from each study and organised thematically to summarise trends in behavioural approach application within different trial-related behaviours and contexts.

**Results:**

A total of 96 studies were included. A majority of these (*n* = 93, 97%) investigated trial conduct processes, such as recruitment and retention. A total of 23 unique theories, models or frameworks were identified. Three behavioural theories, models or frameworks, the Theory of Planned Behaviour (*n* = 23, 24%), Social Cognitive Theory (*n* = 12, 13%) and the Theoretical Domains Framework (*n* = 30, 31%), accounted for more than two-thirds of those utilised. When compared to key stages of the Knowledge to Action cycle, approaches reported in included studies were most often utilised to “Identify a problem” within trials (*n* = 40, 42%).

**Conclusion:**

A wide variety of behavioural approaches to investigate trial conduct were identified. However, the trial processes studied were focused within trial recruitment and largely used a select number approaches. Future research should assess whether these approaches are fit for purpose, as well as prioritising other trial areas for methods research.

**Supplementary Information:**

The online version contains supplementary material available at 10.1186/s13063-025-08808-8.

## Background

Clinical trials remain the gold-standard of evidence for evaluating medical interventions and changes in clinical practice. If conducted rigorously, they provide unbiased estimates of treatment effectiveness, safety and other meaningful clinical outcomes [1]. However, for trials to be conducted rigorously, they themselves need to be informed by evidence on best practice regarding their design, conduct, analysis and reporting [2]. Past research has generated many useful insights and recommendations to inform the conduct of trials, from early considerations as to the need for a trial [3] through to how trial results can reach participants [4]. Unfortunately, translating these insights into meaningful improvements in trial processes has proved difficult [5–7]. Within recruitment and retention to trials (i.e. enrolling and keeping participants on a trial until the end, respectively), systematic reviews of interventions targeting either process have shown a lack of overall effect, focussed replication and quality [8, 9]. A critical oversight in many of these studies could be traced to their lack of a theoretical basis, as recommended when designing such complex interventions [10]. Such theoretical foundations ensure that interventions are designed systematically, with proposed intervention elements being linked to mechanisms to bring about the desired change [11, 12]. They also standardise elements of research language, defining key elements of influence through a shared vernacular, allowing more explicit accounts of what needs to change and how. This shared language also has the benefit of improving dissemination and reproducibility of interventions when its elements and procedures are clearly articulated in terms others are immediately able to understand [11, 12].

There are many theoretical perspectives that one can adopt when investigating trial processes. However, one needs to consider if certain theories can accommodate the complex nature of trials, as well as guiding intervention development [13]. One such field that does meet these requirements is that of behavioural science. Behavioural science defines the actions, people, and contexts that, together, constitute a behaviour and how to go about influencing these behaviours [14]. In a trials context, that means defining the many processes that must be enacted (actions) by the trial staff and the participants (people) at certain places and points in time (context). Use of these behavioural perspectives is present in the trials methods literature, with a prior review identifying a range of theories being applied to investigate and/or intervene on recruitment and retention to trials [15, 16]. Still, this review was limited to those two trial processes, which leaves a significant portion of the trial lifecycle that includes behaviours unaccounted for.

As part of our larger project on incorporating behavioural science into trials methodology, we have conducted a scoping review. Our aim was to identify any instances of a behavioural theory, model or framework (TMFs) being used to investigate any process(es) within clinical trials and/or to develop solutions to target those processes. This review updates and expands the previous review on recruitment and retention by including all processes along the trial lifespan as well as including a wider range of host trials [16].

## Methods

This scoping review was conducted according to a pre-specified protocol (available on the Open Science Framework: https://osf.io/brxcq). The review was conducted to be as systematic in its search and data extraction as possible through adherence to this protocol.

### Types of studies

Eligible studies were set within host clinical trials, making use of their clinical populations to investigate methodological research questions. Clinical trials (hereafter referred to as “trials”) were defined as “any type of research that studies new tests and treatments and evaluates their effects on human health outcomes, including medical interventions, drugs, cells and other biological products, surgical procedures, radiological procedures, devices, behavioural treatments and preventive care” (World Health Organization, 2019). We identified studies that reported research into the development and/or evaluation of a trial:

- Design (i.e. planning the trial design or methods and procedures to be followed for the trial)
- Conduct (i.e. the day-to-day operations of the trial)
- Analysis (i.e. the statistical or other analysis applied to the trial data)
- Reporting (i.e. the dissemination of the trial results and conclusions)

Eligible studies needed to report the explicit use of a TMF in their exploration of any of these trial areas. Trials were either hypothetical or real trials, as defined below. Hypothetical trials are used, broadly, in investigations into the design and/or conduct of proposed trials or identifying general barriers and/or facilitators to trial participation. These investigations are conducted within the same, or similar, populations that a future trial will be targeting for participation.

1. Hypothetical:
  If a study proposes participation* in a general trial based on a disease/condition that potential participants have/could have;
  If a study proposes participation in a specific type of trial (e.g. surgical/pharmaceutical/behavioural) based on a disease/condition that potential participants have/could have;
  If a study proposes participation in a planned trial (i.e. one that is not recruiting);
  If a study proposes participation in an active trial (i.e. one that is recruiting) but is outside the actual trial context (i.e. participants are aware they are not making a real decision to enrol or not);
2. Real—If a study assesses processes in an active/completed trial.

*Participation being any process within a trial but typically recruitment/retention activities

### Inclusion criteria

Types of eligible studies included:

- Those that developed a behaviourally focused understanding of trial processes (e.g. use of semi-structured interviews or surveys developed using behavioural TMFs that identified key domains of importance to trial design/conduct/analysis/reporting);
- Those that developed a behaviourally focused trial process intervention to inform future studies (e.g. empirical studies that have developed interventions, such as using TMFs to develop training packages for health care professionals to improve recruitment to clinical trials, but have not been evaluated);
- Those that have assessed effectiveness of an intervention informed directly by a behavioural TMF that is targeting trial design/conduct versus a comparator (e.g. another intervention or usual practice);
- Explorations/assessments of how the clinical interventions being evaluated in a trial are implemented will be included provided they clearly consider trial processes specific to implementing the intervention within a trial.

### Exclusion criteria

The following exclusion criteria were applied:

- Studies evaluating a trial intervention that did not apply an explicitly defined behavioural TMF;
- Studies exploring the challenges and solutions to poor trial design/conduct that did not use a behavioural TMF to understand findings or develop interventions;
- Studies that aim to improve/evaluate adherence to a clinical intervention being evaluated within a trial rather than a trial-specific process;
- Explorations/assessments of how the clinical intervention(s) being evaluated in a clinical trial are implemented were excluded if they:
  - ◦ Focused on implementing the intervention into clinical practice generally;
  - ◦ Focused on the success/failure of the intervention on the desired health outcome(s).
- Papers that offered commentaries on any trial process without generating primary data and/or analysing data through their chosen TMF;
- Studies in which the context is unclear, i.e. whether the research is being undertaken within a clinical trial context or research in a clinical setting more generally.
- Studies that investigated trial processes alongside other, non-trial research objectives, where we could not reliably separate the results, i.e. the studies report findings as a whole without distinguishing what results are specific to the trial context.

### Search method for identifying studies

A search strategy was designed and refined through discussion with the authors and a Senior Information Scientist (PM) and informed by previous work conducted in this area [8, 9, 16]. Searches were applied to, MEDLINE, Embase, CINAHL, ERIC, PsycInfo, Web of Science and ASSIA from 1966 through November 2023. A search for additional studies was undertaken through a backward and forward citation analysis of three “seed” publications, known to the authors, that met the above eligibility criteria and focused on areas outside recruitment and retention. Additionally, any reviews that met the above criteria for inclusion were screened for primary studies that met inclusion criteria. Finally, identified papers that had developed a trial process intervention informed by a TMF were assessed for published evaluations of said intervention, and those that had evaluated an intervention, a publication of the development was sought.

### Eligibility of studies

Citations identified through the search were independently assessed via title and abstract by two authors, each then screening a select 10% sample of the others to establish consensus. Abstracts were reviewed for explicit mentions of a potential behavioural theory, model or framework, as well as assessing initial eligibility based on the inclusion/exclusion criteria. Any disagreements regarding eligibility were discussed between reviewers to establish consensus with a third member of the team acting to resolve any disputes. Full-text papers were obtained for studies considered potentially relevant and were further assessed against the inclusion/exclusion criteria. The eligibility of a potential TMF as behavioural (based on the definitions below) was determined at this full-text stage. Full-text papers were assessed independently by two authors with a third author helping to establish consensus if there was any disagreement.

### Eligibility of theories, models and frameworks

Authors assessed whether the reported theories, models or frameworks within eligible studies qualified as behavioural through one of two mechanisms. The first was by comparing the theory name, and references for said theory, against a published list of theories of behaviour and behaviour change [14]. This list was developed through expert consensus and sought to identify theories of relevance to public health interventions across social and behavioural sciences [14]. Davis et al. also provided a list of excluded theories that appeared in their search but were agreed to not be behavioural. For TMFs reported in identified studies not explicitly referenced in this list, we then assessed their eligibility through a second mechanism. This second mechanism evaluated whether a TMF met a definition of theory developed by Davis et al., which stated:

“A set of concepts and/or statements with specification of how phenomena relate to each other. Theory provides an organising description of a system that accounts for what is known, and explains and predicts phenomena” [14].

The TMF reported was also evaluated as to whether it considered “individual behaviour as an outcome or part of the process leading to the outcome”, as this review was only concerned with the behaviours of individuals and theories that can explain those behaviours [14]. As such, behaviour was defined as:

“Anything a person does in response to internal or external events. Actions may be overt (motor or verbal) and directly measurable or, covert (activities not viewable but involving voluntary muscles) and indirectly measurable; behaviours are physical events that occur in the body and are controlled by the brain” [14].

Models and frameworks (e.g. Theoretical Domains Framework) were included when we could establish that they had been developed from theoretical constructs from included theories. A model/framework was defined as:

“Organising structures of constructs that do not meet the definition of theory in that they do not offer predictions about how constructs relate to each other or allow prediction of outcomes” [14].

### Data extraction

A data extraction form was piloted and refined by two authors (TC and NY). Information from included studies was extracted by one author and entered into an Excel sheet, with a select 10% sample (sampled to include diversity of trial behaviours, TMFs used, and host trial contexts) assessed by a second author for accuracy and completeness. Examples of data categories included, but were not limited to study design, host trial clinical area/context, study population, trial process category and behavioural approach. National Institute for Health Research guidance on “underserved groups” was used to define these groups reported within included trials [17]. A list of countries that the Organisation for Economic Co-operation and Development (OECD) has defined as developing was used to classify countries under study as “low” or “high” resources settings [18].

### Data analysis

This review aimed to synthesise summary findings across a range of different study designs to describe current research in the area. Results of the studies are presented in a narrative format, separated into sections of thematically similar findings. Quantitative data, such as year and origin of publication, are presented using frequencies.

The TMFs utilised in eligible studies were assessed for how they were applied, being categorised into one of the following three categories derived from the Knowledge to Action (KTA) framework [19]:

1. Identify Problem: Exploratory in nature—may not a priori define the use of the TMF to identify what the barriers/facilitators to trial processes are but uses the TMF more generally to identify or explain one or more trial processes. This phase does not identify targets for change.
2. Assess barriers/facilitators to knowledge use: focuses on targets for change. Uses TMFs to identify behavioural determinants in the context of trial process problems and considers the barriers and facilitators that enhance or dampen these targets. This phase does not include development of interventions to modify the identified targets.
3. Select, tailor, implement interventions: involves TMFs being used to plan and execute interventions to facilitate the design, conduct, analysis or reporting of trials. It involves selecting or tailoring interventions to target the identified barriers. It may consider dissemination (tailoring the message and targeting it to a particular audience) and implementation (systematic efforts to encourage adoption) approaches.

## Results

### Characteristics of included studies

**Fig. 1:**
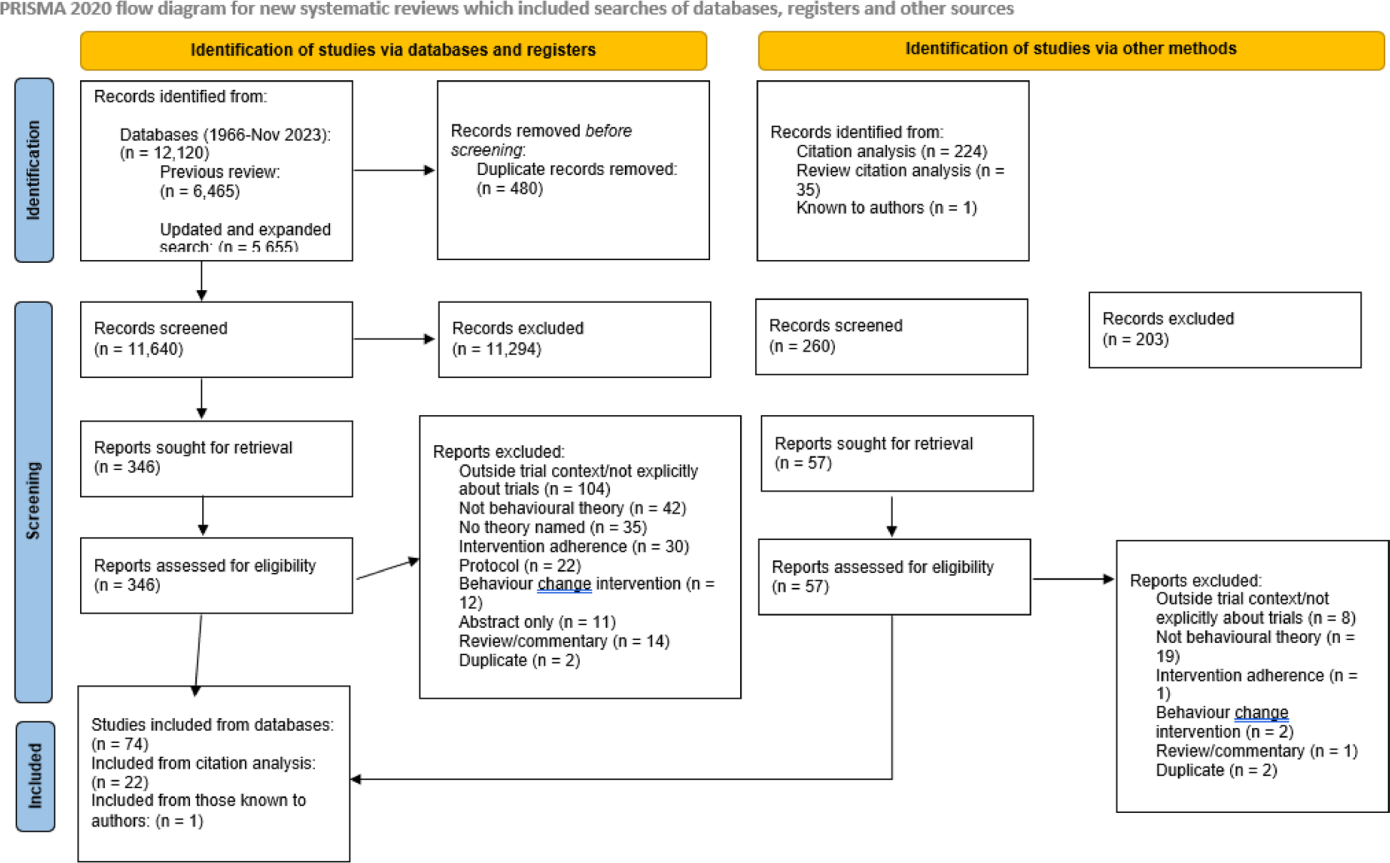
PRISMA diagram

**Fig. 2:**
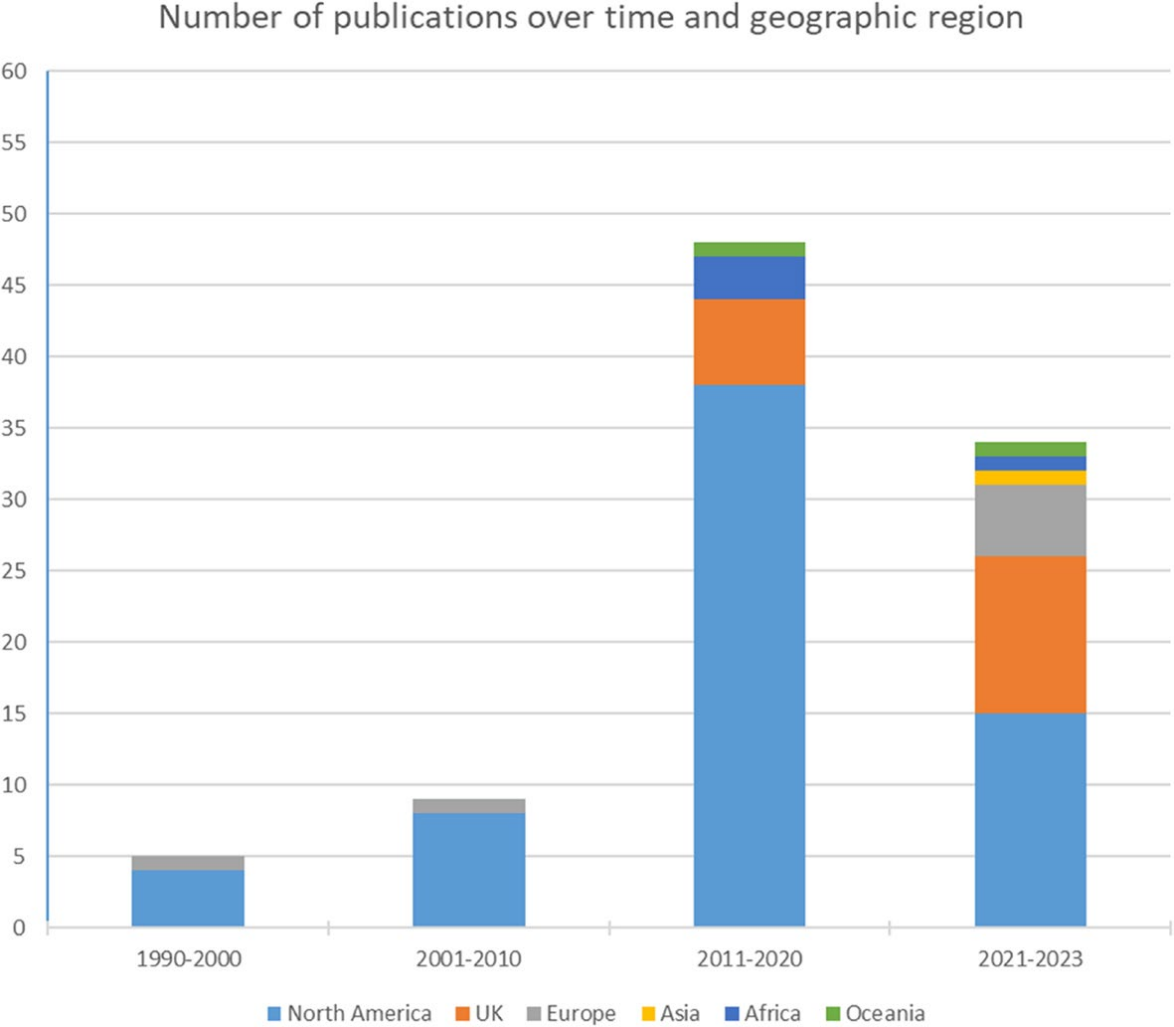
Publications over time and geographic region

A total of 96 eligible publications were identified through our search (see PRISMA diagram in Fig. 1 for more details). A majority of publications (*n* = 48, 50%) were published between 2011 and 2020, and primarily originated from North America (*n* = 65, 68%). A further breakdown of publication year and geographic origin is available in Fig. 2.

### Host trial characteristics

The clinical area of the host trials associated with each publication varied. The most frequently studied clinical areas were cancers (*n* = 26, 27%), followed by HIV/AIDS (*n* = 8, 8%). A subset of publications drew from multiple host trials, either specifying the clinical areas sampled from (*n* = 12, 13%), whilst others did not describe the clinical areas sampled (*n* = 7, 7%). Most of the host trials were evaluating an Investigational Medical Product (such as a drug) (*n* = 24, 25%) or more than one type of intervention (*n* = 21, 22%). Often, the type of intervention was either not stated or there was no specific intervention under study (*n* = 31, 32%). A majority of studies (*n* = 59, 61%) were conducted within “real” trials (i.e. one that participants were actually involved in), whilst the remainder were within a hypothetical trial.

### Demographic characteristics of included studies

Most publications (*n* = 58, 60%) studied participants, potential participants or general members of the public. The remainder studied either trial/clinical staff (*n* = 13, 14%) or a combination of staff and participants (*n* = 25, 26%). The studies were carried out in primarily high resource settings (per OECD criteria) (*n* = 83, 86%). A subset of the included studies (*n* = 42, 44%) explored participation of underserved population in trials, such as racial/ethnic minorities or women.

### Trial process characteristics

The vast majority (*n* = 93, 97%) of included studies explored some aspect of trial conduct. Included studies often explored more than one trial process at a time, reflected in the percent totals below adding to more than 100%. Within conduct, recruitment was the most widely studied process (*n* = 76, 79% of all studies) with retention following (*n* = 17, 18% of all studies). Other trial processes reported in conduct included the implementation of the intervention (*n* = 5, 5% of all studies), implementation of the trial generally (*n* = 4, 4% of all studies) or involvement of patient/public involvement (PPI) members (*n* = 1, 1% of all studies). Other processes studied outside conduct were trial design (*n* = 13, 14% of all studies), analysis (*n* = 1, 1% of all studies) and reporting (*n* = 1, 1% of all studies).

### Theory use

**Fig. 3:**
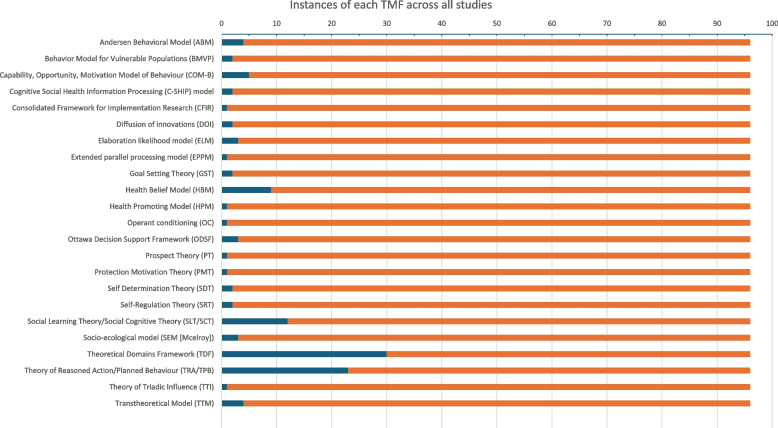
How often each TMF was used in our sample

Most included studies applied the TMFs prospectively (*n* = 85, 89%). The number of individual applications of each TMF is detailed below in Fig. 3 with 23 theories, models or frameworks being reported only once in the included studies. Counts for historically related TMFs (e.g. Theory of Reasoned Action and Theory of Planned Behaviour (TRA/PB)) are combined. Multiple TMFs were reported in some included studies, as such the overall counts of TMFs used exceeds the number of included papers (*N* = 96). The most frequently cited TMF was the Theoretical Domains Framework (TDF) (*n* = 30, 31%), followed by the TRA/PB (*n* = 23, 24%), and Social Learning/Cognitive Theory (SL/CT) (*n* = 12, 13%). Use of these three most frequently cited TMFs varied overtime (see Supplementary Fig. 1). The top three TMFs applied to understand trial behaviours within underserved populations were the TRA/PB (*n* = 13/42), SL/CT (*n* = 9/42) and the TDF (*n* = 5/42) (Supplementary Fig. 2).

### Knowledge to action phases

**Fig. 4:**
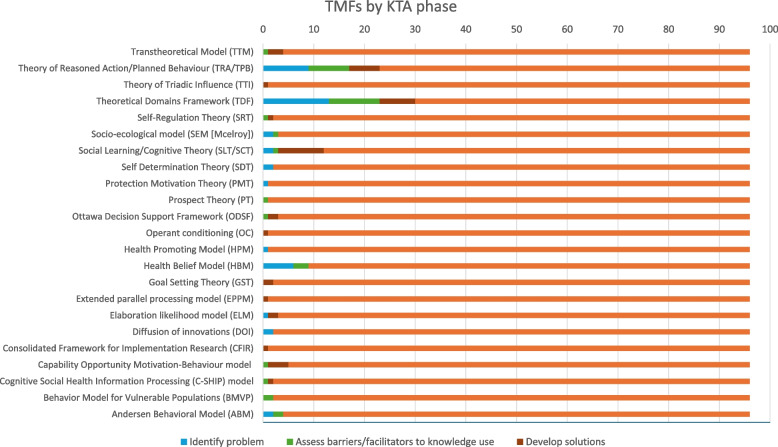
What KTA phase each TMF was used in

In addition to what TMFs were reported and how often, we also assessed how each TMF was applied according to three key KTA phases [19]. Of the 96 included studies, the majority (*n* = 40, 42%) applied the TMFs to “Identify Problem”. These studies were exploratory in nature, utilising the TMFs more generally to probe into aspects of one or more trial processes. The “Identity Problem” studies were often explorations of larger trial processes like recruitment rather than specifying specific behaviours within the overall process, e.g. identification of patients or seeking/providing consent. The other two KTA phases, “Assess barriers/facilitators to knowledge use” and “Select, tailor, implement interventions”, were represented equally in the included studies (28 studies each, 29%). The former focused on more specific targets for change, identifying behavioural determinants for discrete trial processes. Although the “Assess barriers/facilitators to knowledge use” studies stopped short of developing interventions, in some cases they proposed potential strategies to enhance facilitators and reduce barriers to their target behaviours. The third KTA phase, “Select, tailor, implement interventions”, was most often represented by studies applying the selected TMFs to plan and execute interventions targeting trial processes. This could include adapting existing interventions or evaluating whether interventions successfully influenced the behavioural determinants targeted. In some studies, TMFs were applied across all three KTA phases (e.g. TDF used across all three phases), whilst others were applied to one or two of the phases. The mapping of TMFs across the KTA phases is presented in Fig. 4 below.

### Case studies

To further illustrate the application of behavioural TMFs to behavioural challenges of trial design, conduct, analysis or reporting, we have presented six included studies to act as illustrative case studies. These have been purposively selected for variability on their origin, design, population, trial process studied, the TMF reported and how it was applied. These case studies highlight the diverse populations and behaviours of interest being investigated across the studies included in this review (see Table 1).

**Table 1:** Selected case studies from review to illustrate variability of sample

**Citation**	**Aim**	**Process**	**TMF**	**KTA Phase**	**Findings**	**TMF utility**
Mukherjee et al.	"It aims to identify contextual factors that may influence operationalising complex interventions, provide information about adaptations which need to be made to address emergent challenges, and address knowledge gaps on implementation barriers of health service interventions in Low and middle-income countries LMICs."	Design	SEM	Identify problem	“Key challenges included delays in government approvals, addressing community health worker needs, and building trust in the community. These were addressed through continuous communication, leveraging support of relevant stakeholders, and addressing concerns of community health workers and community. Issues related to use of digital platform for data collection were addressed by a dedicated technical support team.”	“The SEM provided the analytical framework to analyse these challenges. We found it to be a useful analytical tool as it provided a comprehensive and layered understanding of how contextual challenges that emerge, need to be addressed at multiple levels.”
Meekes et al.	“This study aims to explore barriers and facilitators regarding recruitment and retention of older adults living in Assisted Living Facilities to a randomised controlled trial study that aimed to improve physical function by using technology. “	Recruitment/ Retention	HBM	Identify problem	“Scheme Managers, therapists, researchers and older adults' peers appear to play an important role in the recruitment and retention of older adults living in Assisted Living Facilities. Additionally, the technology itself and the presentation of the research appear to influence recruitment. Creating a social setting, inviting people face-to-face, demonstrating the technology, showing the benefits by presenting results from a pilot study and alleviating people's fears were experienced as important factors for recruitment.”	“The explored factors that influence the recruitment and retention of the older adults are related mainly to the concepts, Perceived Benefits versus. Perceived Barriers and Cues to Action of the HBM.”
van Sasse et al.	“The aim of this study is to explore to which extent the self-determination theory of Ryan and Deci, according to the ECT (early clinical trial) enrollment phase, corresponds to the motivations of participants during ECT’s.”	Retention	SDT	Identify problem	“Participants felt they tried everything and that they were treated to the limit. This not only gives the motivation to continue participating but also a sense of altruism. Despite different burdens, side-effects, and the feeling of being a test subject, the participants will not easily choose to stop participation in order to prevent saying afterwards: ‘If only I had’.”	SDT guided data collection and analysis. Authors identified themes consistent with each construct in SDT and described each construct’s influence on motivation to participate.
Curran et al.	“Our study was a proof of concept study to operationalize the TDF to conduct a retrospective theory-based process evaluation. More specifically, we wanted to determine whether the domains in the TDF could help explain participants’ lack of response to the strategies employed in the Canadian CT Head Rule trial.”	Intervention implementation	TDF	Assess barriers/ facilitators to knowledge use	“Barriers likely to assist with understanding physicians’ responses to the intervention in the trial were identified in six of the theoretical domains[…]. Despite knowledge that the Canadian CT Head Rule was highly sensitive and reliable for identifying clinically important brain injuries and strong beliefs about the benefits for using the rule, a number of barriers were identified that may have prevented physicians from consistently applying the rule.”	“Our study findings demonstrate that the TDF can provide useful information about behavioural determinants that might aid in post-hoc interpretation of the results of a trial.”
Chalela et al.	"[...]this pilot study aimed to assess the effect of Choices, a bilingual, multi-component intervention on perceived understanding of clinical trials, agreement with stage of decision readiness (stages of change) statements, and consideration of clinical trials as a treatment option among Latina breast cancer patients."	Recruitment	SCT	Develop solutions	“Computer-based videos and care coordination provided by patient navigation—specifically tailored to Latinos—are effective strategies to successfully address awareness, and improved decision-making skills to make informed decisions about clinical trial participation.”	“When comparing the study’s two groups, the Choices pilot trial showed that our theory-based multicomponent intervention was more effective than the control (receiving the NCI fact sheet) in increasing agreement with perceived understanding of clinical trials and consideration of a clinical trial as a treatment option.”
Goulao et al.	"We aimed to find out if trialists involve patient and public partners in numerical aspects of trials and what are the barriers and facilitators to doing it."	Analysis /Reporting (and Design/ Conduct)	TDF	Identify problem	“We found lack of knowledge, trialists’ perception of public and patient partners’ skills, capabilities and motivations, scarce resources, lack of reinforcement, and lack of guidance were barriers to involving public and patient partners in numerical aspects of trials. Positive beliefs about consequences were an incentive to doing it.”	TDF guided data collection and analysis. Authors identified themes consistent with each construct in TDF and one outside the TMF. The TMF structured findings and was perceived as useful for categorising barriers/facilitators to aim..

## Discussion

Our review identified various applications of behavioural TMFs within the context of design, delivery, reporting and analysis of clinical trials. Most of those applications have been within trial conduct, specifically recruitment and (to a lesser extent) retention. Several of the included studies have focused on trials within particular disease areas (i.e. cancers and HIV/AIDs) or trials within underserved populations (e.g. ethnic/racial minority groups, women). Many of the included papers reported well-known behavioural TMFs, such as the Theory of Planned Behaviour and Social Cognitive Theory. However, 23 TMFs were distinct in their application, highlighting a diversity of approaches applied to trials methodology. Most studies sought to “Identify Problems” in their applications of TMFs.

### Areas of investigation

Trial conduct was the primary process category reported within our included studies. A vast majority focused on recruitment and retention to trials, with recruitment research far outpacing retention. This abundance of recruitment-focused research is consistent with findings from previous reviews, both within and outside behavioural science applications [8, 9, 16, 20]. However, efforts to ensure research on retention are also offered appropriate attention has gained traction in recent years [8, 21–23]. Outside of conduct, trial design, the planning of the trial, was the next most frequently studied area, but still small in scale in comparison to the study of trial conduct processes, recruitment and retention.

In terms of clinical area, those studying trials within cancers were the majority. These findings may not be surprising given cancer is a particularly difficult clinical area to conduct trials within but also an area with several committed funding streams. One analysis of nearly eight thousand cancer trials found that 39% of these failed to complete due to poor recruitment [24].

Trials suffer from a lack of diversity in their sample populations, an issue that carries ethical and scientific consequences to the validity of trial results [25]. Recent efforts to correct this imbalance are important steps forward but may lack demonstrable impact if they fail to meaningfully improve both the number and experiences of underserved participants within trials [25]. Our results reflect a significant portion of behaviourally informed methods research focused on this area. Behavioural TMFs applied in the studies typically accommodate multiple socio-ecological levels in their constructs, as such they may be particularly well-suited to explore the complex psychological and socio-economic factors influencing poor participation from underserved groups [13, 14, 26]. Many of the investigations identified in our sample went past exploratory work into the development and testing of interventions aimed at improving underserved groups’ participation.

A number of included studies applied TMFs to develop behaviour change interventions, representing the “Select, tailor, implement interventions” phase of the KTA cycle. Despite some interventions demonstrating effectiveness in improving trial processes like recruitment, they are rarely replicated—a generic issue in the improving recruitment and retention space [8, 9]. It is difficult to ascertain why so many potentially promising interventions appear to fade into obscurity past these initial publications. It may be that any improvements were seen as minimal, particularly when considering the fiscal and opportunity costs associated with further refining and/or implementing these interventions [27]. Another scenario may be that these interventions are implemented, but only within the limits of the trials units that participated in their development. As with clinical improvements, methodological improvements are slow to reach practice and require that their developers promote their implementation to wider user bases, along with the “buy-in” from these user bases that motivates them to adopt innovations [6, 7]. It is essential that these interventions are replicated and evaluated for acceptability and feasibility with trialists. The grounding of interventions in behavioural TMFs can facilitate this work, anchoring their mechanisms and outcomes in shared theoretical language [12, 28].

Finally, most of the included studies were hosted within “real” trials, gathering insights from participants and/or staff from active or completed trials rather than asking about a proposed or otherwise hypothetical trial. This is notable because of the literature available that suggests hypothetical decisions/behaviours can be different from actual behaviour [29, 30]. To frame that within trials, there are likely different theoretical considerations to explain/predict the behaviour of individuals that are faced with the actual consequences of enrolling/conducting a trial versus when they are contemplating it as a possibility. Care should then be exercised when designing/interpreting investigations of hypothetical versus real behaviours within trials, particularly whether mechanisms will generalise to real-world trial processes [30, 31].

### Theory use

The top three TMFs reported in our sample were the Social Cognitive Theory (formerly Social Learning Theory), the Theoretical Domains Framework and the Theory of Planned Behaviour (formerly the Theory of Reasoned Action). The representation of these three TMFs is similar to our past review on application of behavioural science to recruitment and retention and appears consistent with the TMFs most often used in research on healthcare behaviours [14, 16, 26]. Although these TMFs are well-represented in the literature, it is still important to interrogate if these TMFs are accurately and cohesively representing the behavioural influences and mechanisms of clinical trial processes. How to go about choosing a TMF is a challenging endeavour, and one that is often predicated on the experience of the researchers, what they are familiar with, and what has been used previously [12, 13, 26]. This can lead to perpetualisation of TMFs without proper assessment of their fit for purpose or whether their use leads to improved effectiveness in interventions [12, 13]. Even if TMFs are only applied in exploratory roles, some are lacking in their abilities to frame factors outside of individual actors [26]. In complex systems, like trials, it is imperative to incorporate all interacting components within a system that can exert an influence on an individuals’ behaviour [12, 13]. Ideally, all aspects of a specified behaviour should be explicable through a chosen TMF and all constructs of a TMF found to contribute to these explanations [12]. When work moves into developing interventions, the proposed mechanisms of change for the intervention should be explicitly linked to the explanatory constructs of the TMF [13, 26, 31]. Further, any evaluation of an intervention’s effectiveness should select outcome measures that assess whether the appropriate theoretical constructs were influenced, via the proposed mechanism of action, leading to the desired change in behaviour [11, 26, 32–34]. Many of the included studies within our review do not clearly report information about theory selection, application or evaluation, if at all. This often led to confusion in when a TMF was applied, how much of it was applied, and what trial-related behaviours researchers were trying to target [12, 32]. Without proper specification of the target behaviour, it is unclear to those outside the research team what explicit behaviours within larger conceptual processes, such as “recruitment”, “participation” or “enrolment”, are being targeted in the investigation. Such ambiguity deters attempts to collate findings across papers, except at the highest level of processes [28, 35]. With so many individual, distinct behaviours existing within processes like recruitment, collapsing them under one umbrella term fails to illustrate the heterogeneity of behaviours being studied. Lack of specification also precludes attempts to replicate findings/interventions or to adapt interventions to other populations and contexts [11–13, 32, 33]. This lack of reporting clarity is not unique to trials methodology but is particularly burdensome to trialists that do not have the time or possibly experience to tease out essential details on intervention operationalisation, and how they might be able to refine implementation to suit their individual trial centre circumstances [7, 12, 32, 36].

### Strengths and limitations

This review has several strengths. Our search strategy was designed to be as inclusive as possible, of both behavioural TMFs and trial processes. The volume of results returned from the database search makes it unlikely that much published literature was missed. Incorporation of a forward–backward citation analysis further reduces this possibility. One potential limitation to our search strategy is the search terms utilised to capture processes outside of conduct. The review utilised search terms generated for previous reviews of recruitment and retention research but did not benefit from similar resources outside those areas. Instead, we added search terms based on key papers within trial processes outside of recruitment and retention to ameliorate this potential gap.

The review benefitted from prior work to identify behavioural TMFs and we referenced their results extensively when including TMFs [14]. We also utilised Davis et al.’s definition of theory and applied it to potential TMFs outside their list, further ensuring all relevant TMFs were captured [14]. This process, however, relied on checking the provided references for the TMF cited in the included study in order to verify if the criteria for behaviour and theory were met. There were some instances in which the cited TMF references were insufficient to make an informed decision on the TMFs inclusion and so were excluded.

### Conclusions and future directions

This work has highlighted the wide-ranging application of behavioural science to processes within clinical trials. However, it also reinforces a focus of trials methods research being on recruitment and retention. Future activity should consider other trial related behaviours, such as dissemination of trial results to participants, when employing a behavioural lens. Efforts to standardise how behaviourally focused research is conducted and reported are ongoing and need to be implemented within the trials methodology space [12, 35, 37]. This will be essential in synthesising evidence on factors underlying trial participation, and related processes, and how best to improve it. Further clarity in TMF application will allow for hypothesis testing of theoretical constructs, either providing evidence of fit or demonstrating lack of utility, both providing a more succinct repertoire of potential TMFs to choose from in future studies [26, 38]. Ongoing research aims to provide agreement on which TMFs can be considered further for their utility in understanding trial processes. Further, the theoretical constructs of some behavioural TMFs can be linked with “active ingredients”, known as behaviour change techniques (BCTs), in behaviour change interventions [39]. Appropriate BCTs can be selected based on the theoretical constructs that need to be targeted, such as the “Instruction on how to perform the behaviour” BCT being utilised in an intervention to improve staff recruitment practices through consent training. The updated and expanded collection of BCTs is now available alongside other essential intervention components, such as “Mode of Delivery”, within the Behaviour Change Intervention Ontology (BCIO). The higher level of description afforded by the BCIO will allow intervention developers to specify precisely how their interventions are meant to work, how they are meant to be delivered and other essential information for adaption, replication and syntheses of interventions and their effects [40]. Extending the methodological application of behavioural TMFs across trials (and processes) in a range of settings, recruiting diverse populations and testing a variety of interventions will further develop the knowledge base on how behavioural science can be leveraged to improve clinical trials by making their design, conduct, analysis and reporting more evidence based and participant centred.

## Supplementary Information

Supplementary Material 1

Supplementary Material 2

## Data Availability

An abbreviated, live version of the included studies in the review, along with PDF copies of those included studies, are available on the Open Science Framework (https://osf.io/zkpv4/?view_only=01faeffd2c904496945c46c33f26d841). All other datasets used and/or analysed during the current study are available from the corresponding author on reasonable request.

## Abbreviations

BCIO: Behaviour change intervention ontology
BCT: Behaviour change technique
KTA: Knowledge to Action
PPI: Patient-public involvement
PRISMA: Preferred Reporting Items for Systematic Review and Meta-Analyses
OECD: Organisation for Economic Co-operation and Development
SL/CT: Social Learning/Cognitive Theory
TDF: Theoretical Domains Framework
TMFs: Theory, model, or framework
TRA/PB: Theory of Reasoned Action/Planned Behaviour
