# Supplementary material for: A scoping review of how behavioural theories, models and frameworks have been applied to the design, conduct, analysis or reporting of clinical trials
Source: Trials. 2025 Mar 25;26:104. doi: 10.1186/s13063-025-08808-8 (PMC11934719; doi:10.1186/s13063-025-08808-8)
Supplement: Supplementary file 1 — Supplementary Material 1 [file 13063_2025_8808_MOESM1_ESM.pptx]

## Slide 1
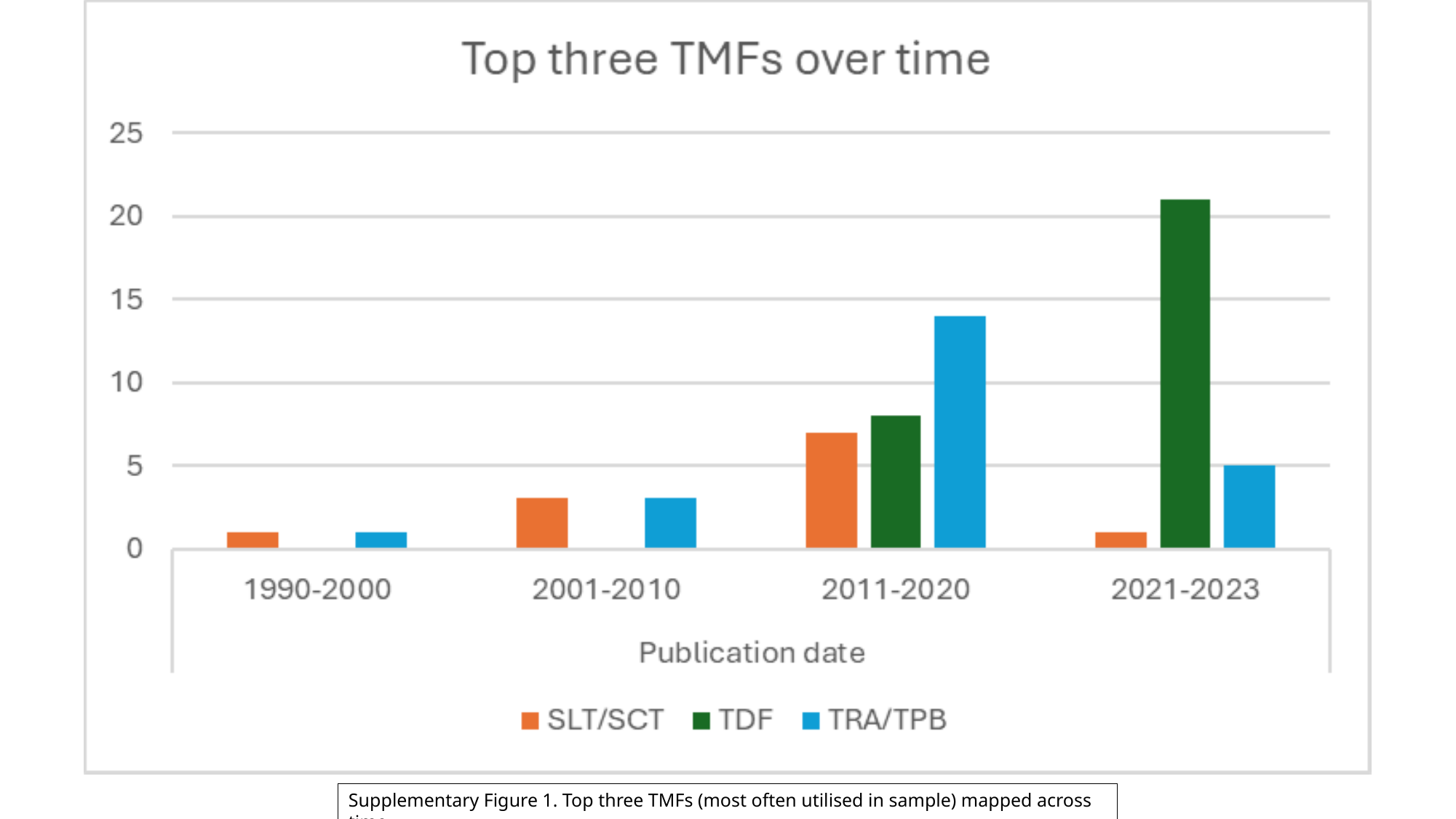

Supplementary Figure 1. Top three TMFs (most often utilised in sample) mapped across time

## Slide 2
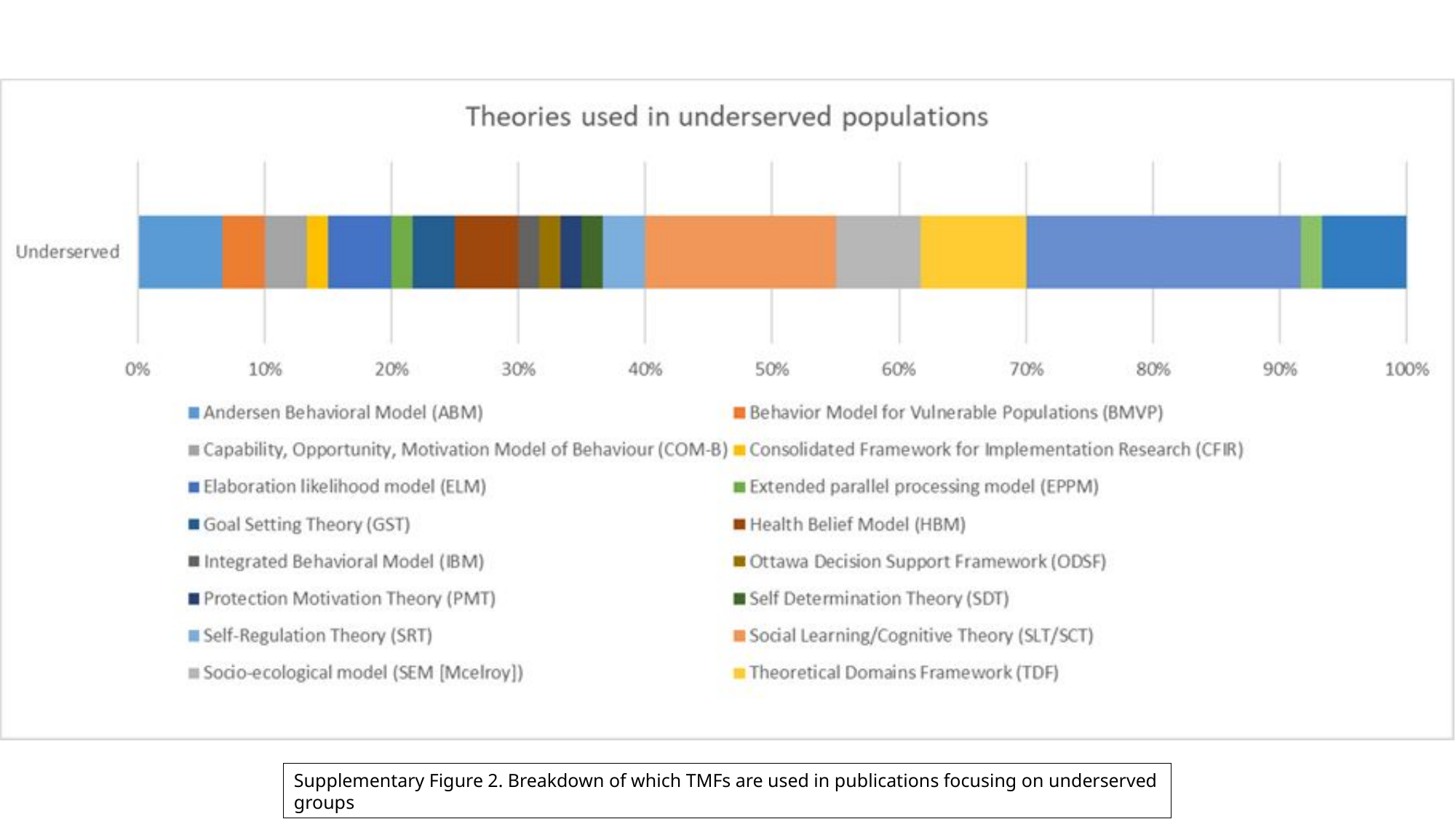

Supplementary Figure 2. Breakdown of which TMFs are used in publications focusing on underserved groups
